# An overview on the interplay between nutraceuticals and gut microbiota

**DOI:** 10.7717/peerj.4465

**Published:** 2018-03-13

**Authors:** Adrian Catinean, Maria Adriana Neag, Dana Maria Muntean, Ioana Corina Bocsan, Anca Dana Buzoianu

**Affiliations:** 1 Department of Internal Medicine/Faculty of Medicine, University of Medicine and Pharmacy of Cluj-Napoca, Cluj-Napoca, Romania; 2 Department of Pharmacology, Toxicology and Clinical Pharmacology/Faculty of Medicine, University of Medicine and Pharmacy of Cluj-Napoca, Cluj-Napoca, Romania; 3 Department of Pharmaceutical Technology and Biopharmaceutics/Faculty of Pharmacy, University of Medicine and Pharmacy of Cluj-Napoca, Cluj-Napoca, Romania

**Keywords:** Nutraceuticals, Firmicutes, Microbiota, Bacteroidetes

## Abstract

**Background:**

Nowadays, growing attention was being given to the alternative ways to prevent or treat diseases. Nutraceuticals are used increasingly for this purpose. Many of these are being used as alternative therapy. Classic therapy with synthetic drugs, although very effective, has many side effects. The term “nutraceuticals” refers to the link between the nutritional and pharmaceutical domains. Also, lately, many studies have been done to investigate the role of microbiota in maintaining health. There is the hypothesis that some of the health benefits of nutraceuticals are due to their ability to change the microbiota. The aim of this review was to emphasize the link between the most commonly used nutraceuticals, the microbiota and the health benefits.

**Methods:**

We selected the articles in PubMed, published up to July 2017, that provided information about most used nutraceuticals, microbiota and health benefits. In this review, we incorporate evidence from various types of studies, including observational, *in vitro* and *in vivo*, clinical studies or animal experiments.

**Results:**

The results demonstrate that many nutraceuticals change the composition of microbiota and can interfere with health status of the patients.

**Discussion:**

There is evidence which sustains the importance of nutraceuticals in people’s health through microbiota but further studies are needed to complete the assessment of nutraceuticals in health benefit as a consequence of microbiota’s changing.

## Introduction

The “nutraceuticals” terminology was firstly introduced by Dr. Stephen L. DeFelice in 1989 and represents the link between nutrition and the pharmaceutical field. After 1994, when the place of dietary supplements in maintaining people’s health was established, the term “nutraceuticals” was extended to include this category, too. Vitamins, minerals, herbs, amino acids and others belong to dietary supplements class, hence to the nutraceutical class (Gupta et al., 2010). Nutraceuticals are products isolated or purified from foods. They are sold in dosage form and are not usually associated with food, which play an important role in modifying and/or maintaining physiological functions or offering protection against chronic diseases (Das et al., 2012; Romano et al., 2012).

Nutraceuticals can be classified in *potential* nutraceuticals and *established* nutraceuticals. The majority of nutraceuticals belong to the first category, as many preclinical and clinical studies are still necessary in order to prove their beneficial effects. Only a few classes belong to the second category, e.g., probiotics, prebiotics, omega 3 fatty acids and antioxidants. For these classes, many studies which confirm beneficial effects in certain conditions are already available (Pandey, Verma & Saraf, 2010; Alisi et al., 2014; Riva et al., 2017).

Furthermore, there are two other classifications of nutraceuticals: based on chemical constituents (Nutrients, Dietary Supplements, Herbs) or Traditional/Non-traditional nutraceuticals (Gupta et al., 2010).

We cannot refer to nutraceuticals without connecting them with the human’s microbiota which represents the total microorganisms which colonize the host at different levels: gastrointestinal tract, respiratory tract, skin, vagina and so on. The number of these microorganisms is estimated to 10^14^, representing a number 10 times higher than that of human cells (Thursby & Juge, 2017).

The most developed one is the gastrointestinal microbiota with an unequal distribution along the digestive tract. There are few bacteria in the stomach and first part of the small intestine, but the concentration increases reaching a maximum in the colon (Montalto et al., 2009).

Adult healthy subjects have six bacterial phyla dominating the gut microbiota: *Firmicutes* and *Bacteroidetes* (90%), *Proteobacteria, Actinobacteria, Fusobacteria* and *Verrucomicrobia* (Woting & Blaut, 2016).

*Firmicutes* and *Bacteroidetes* represent the main bacteria phyla, whose proportion remains the same during lifetime for a person (older than 3 years old) and their family members (Tang & Hazen, 2014).

The aim of this review is to summarize the current knowledge about the nutraceuticals interaction with gut microbiota and how we could use this class of supplement in different pathologies related to dysbiosis and leaky gut condition. Furthermore, there will be listed a number of nutraceuticals scientifically proved to have beneficial effect on healing the gut barrier and promoting the eubiosis.

## Survey Methodology

We performed an electronic literature search in the PubMed database and we included relevant articles published after 2000. In our search we used the following terms: “nutraceuticals”, “food supplements”, “health benefits” in combination with “gut microbiota” or “gastrointestinal bacteria”. In this review, we included evidences from various types of studies: observational and experimental studies, both *in vitro* and *in vivo* researches, including randomized controlled ones. Overall selected papers were used for the purposes of this review.

### The importance of gut microbiota

A few species of the colon’s bacteria have an important role in the bacterial degradation of amino-acids and formation of short-chain fatty acids (SCFAs)—rich sources of energy for the host and metabolic signaling (Jandhyala et al., 2015; Woting & Blaut, 2016). *Bacteroidetes* produce acetate and propionate, while *Firmicutes* families (*Lachnospiraceae, Ruminococcaceae*) mainly produce butyrate as primary metabolic end products (Den Besten et al., 2013; Louis & Flint, 2017). Butyrate is the most important component of energetic metabolism in the colonocytes, while acetate and propionate are used as substrates for lipogenesis, gluconeogenesis and protein synthesis (Schwiertz et al., 2010; Tremaroli & Bäckhed, 2012). Acetate, propionate and butyrate play an important role in the regulation of hepatic lipids and glucose homeostasis, including the effect of peroxisome proliferator-activated receptors (PPAR) on gluconeogenesis and lipogenesis (Morrison & Preston, 2016).

Moreover, the gut bacteria metabolize tryptophan to active substances. For example, *E. coli* produces indoles which play an important role as signaling molecules with mainly physiological roles (motility, biofilm formation and antibiotic resistance) (Li & Young, 2013; Levy, Blacher & Elinav, 2017).

The SCFAs inhibit histone deacetylase (HDAC) and ligands for certain G-protein coupled receptors (GPRs). The result of broad expression inhibition of both (HDAC and GPR) explains the physiological roles of SCFAs: regulation of blood pressure, kidney function, nervous system and protection against colon cancer (Alex et al., 2013; Pluznick, 2014; Steinmeyer et al., 2015; Joseph et al., 2017).

SCFAs are also involved in the activation of free fatty acid receptors—FFAR2 and FFAR3—G protein-coupled receptors that function as signaling molecules in many physiological processes. These receptors can be found in many cells: adipocytes, pancreatic islet, incretin-releasing enteroendocrine cells (K cells—gastric inhibitory polypeptide release, I cells—cholecystokinin release, L cells—glucagon-like peptide-1 and peptide YY release). Moreover, FFAR2 are expressed in neutrophils and small intestine dendritic cells (Hara et al., 2011; Alvarez-Curto & Milligan, 2016).

**Figure 1:**
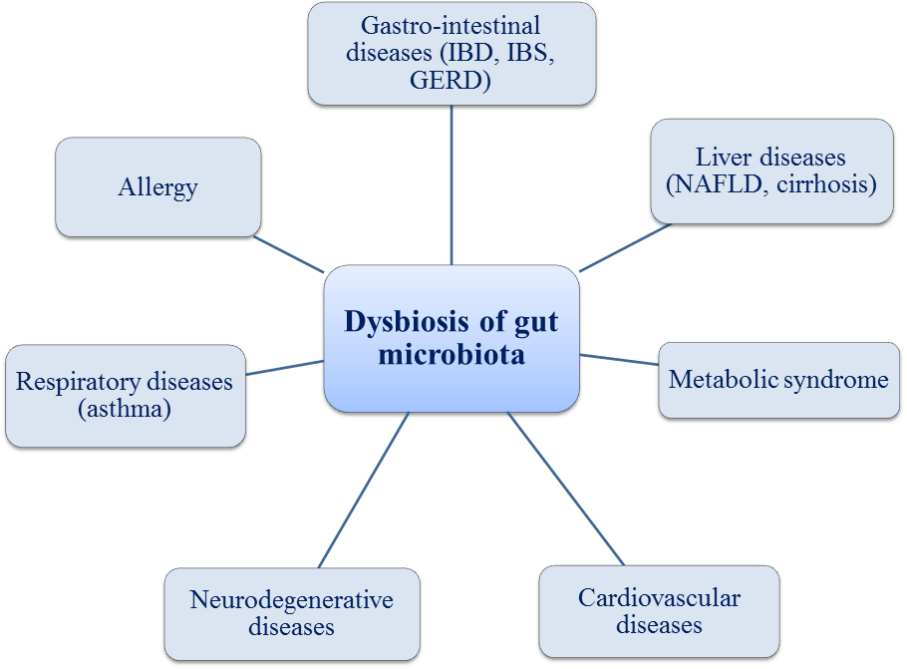
Gut microbiota and associated health problems.

**Table 1:** The link between several diseases and changes of microbiota.

Disease	Changes in microbiota’s diversity and composition	Consequences	Reference
Inflammatory bowel disease	Less bacterial diversity ↓ the number of *Bacteroides* and *Firmicutes*	decreasing the concentration of butyrate	Lucas López et al. (2017)
Irritable bowel syndrome—diarrhea	↑*Enterobacteriaceae*↓*Faecalibacterium prausnitzii*	not known	Dupont (2014)
Constipation	↑*Firmicutes(Lachnospiraceae and Ruminococcaceae)*↓*Bacteroidetes (Prevotella)*	increasing the production of butyrate	Zhu et al. (2014)
Obesity	Changes in the ratio of *Bacteroidetes/Firmicutes*↓ the abundance *Akkermansia muciniphila*↑ the abundance *Campylobacter*, *Shigella, Prevotella*	decreasing the production of butyrate	Festi et al. (2014)Tremaroli & Bäckhed (2012)
Diabetes tip 2	↓*Bifidobacterium spp* significant association of *Parabacteroides* with diabetic patients	not known	Wu et al. (2010)
	↓*Firmicutes*↑*Bacteroidetes, Proteobacteria*	it is possible to determine endotoxemia → oxidative stress → IL1, IL6, TNF *α*	Marlene (2013)
Diabetes tip 1	↓*Lactobacillus, Bifidobacterium, Blautia coccoides–Eubacterium rectale, Prevotella*	decreasing the production of butyrate decreasing the synthesis of mucin increasing the intestinal permeability	Murri et al. (2013)
	↓*Clostidium clusters IV and XIV (species that produce butyrate)*	decreasing the production of butyrate	De Goffau et al. (2014)
Dyslipidemia	↓*Lactobacillus*	decreasing enzymatic deconjugation of bile acids → increasing the level of cholesterol	Kumar et al. (2012), Ramakrishna (2013)
Nonalcoholic steatohepatitis	↓*Firmicutes*↓*Faecalibacterium and Anaerosporobacter (order Clostridiales)*↑*Parabacteroides and Allisonella (order Aeromonadales)*	increase in luminal gut ethanol production metabolism of dietary choline release of lipopolysaccharides increasing small intestinal bacterial overgrowth increasing endotoxemia increasing lipopolysaccharide →↑ insulin resistance and ↑ TNF alpha	Compare et al. (2012), Wong et al. (2013) and Machado & Cortez-Pinto (2012)
Acute coronary syndromes	not know	trimethylamine is formed by gut microbiota from nutrients which contain l-carnitine, choline, phosphatidylcholine followed by the formation of trimethylamine N-oxide (TMAO) by hepatic enzymes increasing the plasmatic level of TMAO–increasing the risk of myocardial infarction and stroke	Trøseid (2017)
Autistic spectrum disorders	↑*Clostridium histolyticum (Clostridium clusters I and II)*↑*Bacteroidetes, Desulfovibrio*↓*Firmicutes*	increasing the production of neurotoxins	Parracho et al. (2005), De Angelis et al. (2013)
Allergy	↑*Lactobacillus, Enterococcus*	increasing of allergic sensitization	Kirjavainen et al. (2002)
	low diversity of microbiota ↑*Bacteroidales*↓*Clostridiales*	not know	Hua et al. (2016)

Many studies demonstrated that SCFAs play important roles in the prevention and treatment of the metabolic syndrome, gastro-intestinal diseases (Crohn’s disease, ulcerative colitis or antibiotic-associated diarrhea) and even in certain types of cancer (Den Besten et al., 2013; Kim, Park & Kim, 2014; Weitkunat et al., 2017; Zeng et al., 2017). It is well-known that exposure to various environmental factors (diet, drugs, toxins and pathogens) can cause the alterations of the gut microbiota, known as dysbiosis, with a direct implication in the production of SCFAs. Many studies were dedicated to microbiome and gut barrier. The interaction between microbiota and host immune system proved to be the key element in a new understanding the pathogenesis of a large spectrum of diseases like GI tract disease (inflammatory bowel disease IBD, irritable bowel syndrome IBS, nonalcoholic steatohepatitis NASH, cirrhosis, chronic pancreatitis, gastro-esophagean reflux disease GERD) or extra-intestinal diseases (allergy, asthma, metabolic syndrome, cardiovascular diseases,neurodegenerative disease, psychiatric disease and autoimmune disease) (Fig. 1) (Tang & Hazen, 2014; Carding et al., 2015). However, the microbiota changes over time and there are many variations in diversity and abundance of bacteria between children and the elderly (Buford, 2017; Elderman et al., 2017). The link between several diseases and changes of the microbiota is summarized in Table 1.

**Figure 2:**
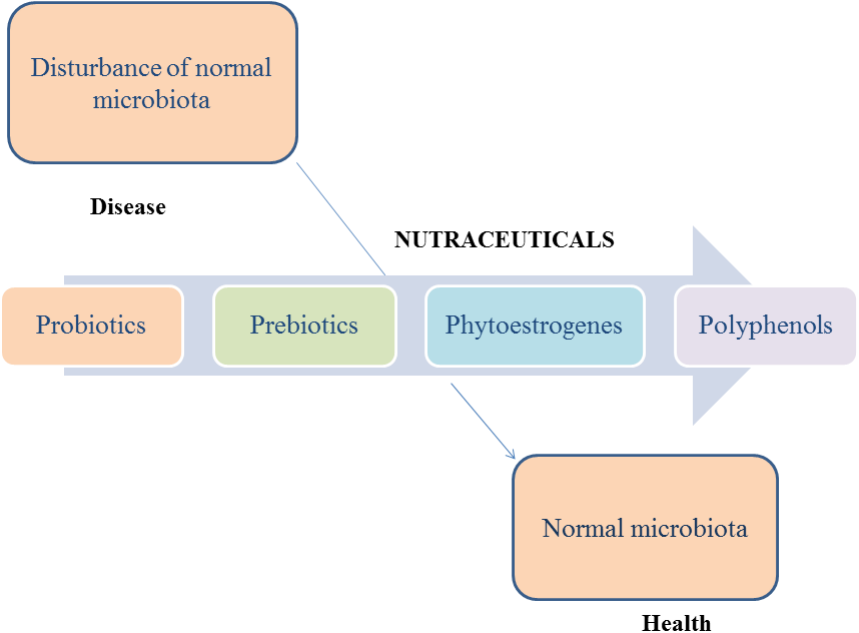
The role of nutraceuticals on microbiota.

According to the latest surveys, there are many reported dietary supplements used to restore the equilibrium of gut microbiota (Fig. 2).

**Probiotics** have been defined by WHO as “live micro-organisms which, when administered in adequate amounts, confer a health benefit on the host” (Walker & Lawley, 2013).

The probiotics can restore the normal composition of microbiota (abundance and diversity) by producing SCFAs. There are many studies which demonstrated that *Lactobacillus* species (*L. casei, rhamnosusand acidophilus*) can prevent or can reduce the severity of antibiotic associated diarrhea (AAD) (Huazano-Garcia, Hakdong & Lopez, 2017; Park et al., 2017), while species belonging to *Lactobacillus, Bifidobacterium* or *Escherichia coli* Nissle 1917 can prevent or treat gastro-intestinal diseases or metabolic disorders (Woting & Blaut, 2016).

Johnston and coworkers performed a meta-analysis study in order to determine whether concomitant administration of probiotics and antibiotics prevents or diminishes the AAD in children. Twenty-three studies were included in this meta-analysis (Johnston et al., 2011). The probiotic treatment was constituted especially of *Bacillus sp, Bifidobacterium sp, Clostridium butyricum, Lactobacilli spp., Lactococcus spp., Leuconostoc cremoris, Saccharomyces spp., or Streptococcus spp.*, alone or in combination. The results demonstrated a beneficial effect of probiotics on AAD incidence (probiotic 8% vs. control 19%) (Johnston et al., 2011).

*Akkermansia muciniphila,* discovered in 2004 by Derrien et al. (2004) is a mucin-degrading bacteria commonly found in the human gut. It represents about 3–5% of the humans’ microbiota. *A. muciniphila* is known for its probiotic properties and its potential benefits in the prevention and treatment of many metabolic disorders (Zhou, 2017). The abundance of *A. muciniphila* was strongly correlated with several parameters (glucose, insulin and leptin) which are the expression of metabolism either lipidic or glucidic and of inflammation (Schneeberger et al., 2015). The bacteria is involved in the modulation of the mucus thickness, gut barrier integrity and immunity, probably due to its localization in the mucus layer, close to the epithelial lays (Ottman et al., 2017; Zhou, 2017). Moreover, *A. muciniphila* plays an important role in the adaptation of the body to cold temperature. It acts as an energy sensor, its abundance increases with caloric deficiency and decreases with excess energy (Chevalier et al., 2015). Modulating the Microbiome —Probiotics hold great promise to modulation the microbiome and confer protection in conditions like metabolic endotoxemia (ME). It is clear that dysbiosis drives ME, thus, a healthy microbiome has the capability to protect the body from ME. The major issue with most probiotics is that they do not survive gastric passage to enter the small or large intestines intact and viable. However, there are spore probiotics that have the capacity to survive the harsh gastric passage and enter the intestines completely viable. As a result, bacterial spores are the only strains that have been shown to treat ME (McFarlin et al., 2017; Zhou, 2017).

**Prebiotics** are nutraceuticals which were approved for bolstering the growth of microbiota’s beneficial species. Carbohydrates are the main source of SCFAs—acetate, propionateand butyrate. Due to the action of SCFAs, the composition and diversity of microbiota can be changed in a positive way (Shashkova et al., 2016). The metabolites decrease the intestinal pH, suppress the intestinal pathogens’ growth and influence intestinal motility (Bron et al., 2017).

The term “prebiotic” refers to “dietary carbohydrates that stimulate the growth of gut bacteria or probiotics when these are administered externally.” A prebiotic is a non-digestible compound that through its metabolization by microorganisms in the gut modulates the composition and/or activity of the gut microbiota, thus conferring a beneficial physiological effect on the host” (Bindels et al., 2015). Fructo-oligosaccharides (FOS), galacto-oligosaccharides (GOS), xylo-oligosaccharides (XOS), lactulose, non-digestible carbohydrates inulin, cellulose, resistant starches, hemicelluloses and pectins are the most used prebiotics in current medical practice (Dahiya et al., 2017; Espín, González-Sarrías & Tomás-Barberán, 2017). The bacterial abundance of gut microbiota is enhanced when a diet is low in prebiotics (Lucas López et al., 2017).

A prebiotic agent must fulfill certain criteria, such as not being hydrolyzed and absorbed in the first part of the gastrointestinal tract and is fermented by a limited number of beneficial bacteria in the colon (e.g., *Lactobacillus*) (Kolida, Tuohy & Gibson, 2002). Moreover, the prebiotic must be able to stimulate or metabolically activate the growth of these beneficial bacteria, in order to change the microflora into healthier one (Kolida, Tuohy & Gibson, 2002). The main effects of prebiotics include modulation of gut microbiota composition and production of energy metabolism, increasing mineral absorption, regulation of immune function and improvement of the intestinal barrier functions (Bron et al., 2017).

Soy beans and lactose from cow’s milk are the main sources of GOS. These are included especially in infant foods. The advantages of GOS are high solubility, neutral taste, stability at high temperature, acidity and low glycemic index. It was demonstrated that if the preterm infants were fed with a combination of 90% GOS and 10% FOS, the composition of gut microbiota increased in *Bifidobacteria* and *Lactobacilli*, resembling breast-fed infants’ microbiota (Sangwan et al., 2011). The mixture of GOS/FOS has a significant impact on increasing *Bifidobacteria* in the gut and on decreasing *Clostridium* but GOS alone has been reported to only increase *Lactobacillus* (Vandenplas, Zakharova & Dmitrieva, 2017).

A randomized, double-blind placebo-controlled study, which included 40 patients divided into two parallel groups (placebo vs prebiotic), demonstrated the beneficial effect of prebiotics (inulin and FOS) on the population of *Lactobacillus* and *Bifidobacteria* for oncologic patients who were treated with pelvic radiotherapy (RT). Stool samples were collected 7 days before the start of RT, 15 days after starting, at the end of RT and 3 weeks after RT was completed. The *Lactobacillus* and *Bifidobacteria* were analyzed and calprotectin level was determined as the marker of intestinal inflammation. The results showed that RT had a negative impact on *Lactobacillus* and *Bifidobacteria* in both groups, however, at 3 weeks after the end of RT, recovery of the gut microbiota was enhanced in patients treated with prebiotics (inulin and FOS) (Velasco et al., 2012). Therefore, inulin and FOS have a beneficial effect on *Lactobacillus* and *Bifidobacteria* populations of the gut.

Onions *(Allium cepa)* are a good source of carbohydrates, vitamins, and minerals. The health benefits of this plant are associated with its chemical components: carbohydrates (FOS and polysaccharides) with prebiotic effect, sulphur compounds (thiosulphinates) and phenolic compounds such as flavonoids (quercetin derivates) (Rodríguez Galdón, Rodríguez Rodríguez & Díaz Romero, 2008).

Filocamo et al. studied the effect of a commercial garlic powder upon pure, commensal bacterial cultures: *Lactobacillus casei* subsp casei DSMZ 20011, *Clostridium nexile* A2-232, *Bifidobacterium longum* DSMZ 20090 and *Bacteroides ovatus*. Results showed that *C.  nexile* and *B. longum* were more susceptible than *L. casei* and *B. ovatum*. *C. nexile* was the most sensitive strain, while *L. casei* was the least affected. Other studies demonstrated that garlic has bactericidal effect against *E. coli*, *Salmonella typhimurium*, *Neisseria gonorrhoeae*, *Staphylococcus aureus* and *Enterococcus faecalis* (Filocamo et al., 2012; Santhosha, Jamuna & Prabhavathi, 2013).

Aloe vera (*Aloe barbadensis* Miller) contains several compounds (anthraquinones, carbohydrates, vitamins, minerals, enzymes, amino acids) with many beneficial effects. Moreover, it is considered an important and natural source of prebiotics. It was observed that the leaf gel, due to oxidation, leads to fermentation and determines bacteria growth. Several research groups reported lactic acid bacteria after the fermentation of *Aloe vera* pulp, laying the grounds for studying the probiotic effect of *Aloe vera*. Five *Lactobacillus brevis* strains were isolated from naturally fermented *Aloe vera* pulp and the properties of a good probiotic were highlighted, such as high tolerance to acid (surviving 4 h in pH = 2.5) and the inhibition of the development of many enteropathogenic bacteria (*C. jejuni, C. perfringens, S. aureus, Salmonella, E. coli)* (Kim et al., 2014; Cuvas-Limón et al., 2016; Chiodelli et al., 2017).

Not only prebiotics and probiotics may influence gut microbiota. There are also other nutraceuticals, like phytoestrogens, polyphenols, etc. that may play a significant role in compounds that were isolated and characterized from them. The other nutraceuticals with benefic effect on gut microbiota are presented below.

**Phytoestrogens** are natural compounds with structural and functional similarities with estrogen hormones. These compounds are classified in: flavonoids, isoflavonoids, lignans, ellagitannins, coumestansand stilbenes (Bilal et al., 2014; Gaya et al., 2016). A single plant can contain many types of phytoestrogens and different parts of plants can contain different amounts of phytoestrogens (Jarošová et al., 2015). The main sources for phytoestrogens are fruits, vegetables and whole grains (Sirotkin & Harrath, 2014).

Flavonoids are transformed by bacterial species in the intestine, but they can also represent a substrate for the human gut microbiota. This can influence the absorption at intestinal level. Flavonoids influence and regulate the intestinal barrier and intestinal permeability and it was demonstrated that they have a direct trophic influence on *Akkermansia* and not on mucin production (Cassidy & Minihane, 2017).

Flavonoids possess antimicrobial effect, offering protection against pathogenic bacteria, fungi and viruses. In this era of antimicrobial resistance, the flavonoids could be considered suitable alternatives to antibiotics, especially in mild/moderate infections or in prevention (Iranshahi et al., 2015).

Anthocyanins belong to the flavonoids’ class and can be found in berries, grapes, apples etc. These compounds have an important role in the prophylaxis of many cardio vascular and neurological diseases, metabolic disorders and even cancer, especially due to their antioxidant properties (Chaiyasut et al., 2017). Berries are known for their high content in anthocyanins. The supplementation of mice’s diet with blueberrys or blackcurrants determined a significant change in gut microbiota, by promoting the anaerobic bacteria *Bacteroidetes* and *Actinobacteria*, probably through their antioxidant effect (Overall et al., 2017).

The isoflavonoids are a subclass of flavonoids, polyphenols, which is in high amount in soy, soy proteinand miso. The main sources of lignans are flaxseeds, soybeans, strawberries, carrots, cabbage, onion, garlicand cucumber (Blanck et al., 2003; Peterson et al., 2011).

The isoflavones are not active in primary form, as conjugate glucosides. The gut microbiota and intestinal mucosa have an important role in the conversion of these glucosides in compounds which are well absorbed and metabolized by the intestinal microflora into other metabolites (Vitale et al., 2013). These metabolites have estrogen-like activity (Laparra & Sanz, 2010). Isoflavonoids contain many glycosides, such as genistein, daidzein and glycitein. Daidzein is metabolized by gut bacteria to equol (Franke et al., 2014), which plays an important role in the health benefits of soy and it has strong estrogenic activity and antioxidant capacity. The main bacteria that contribute to the conversion of the isoflavonoids into equol inhabit the distal portion of the gut and belong to the family *Coriobacteriaceae* (Guadamuro et al., 2017).

Furthermore, along with isoflavonoids, ellagitannins and lignans are metabolized by gut bacteria into equol, urolithins and enterolignans, which have high bioavailability and estrogenic/antiestrogenic effects, antioxidant, anti-inflammatory and antiproliferative effects (Gaya et al., 2016).

**Polyphenols** have intensively been studied in the last years due to their beneficial effects in both cardio-vascular diseases and cancer. They are very expressed in our diet. The main sources of polyphenols are cocoa powder, dark chocolate, berries, beans, nuts, vegetables (red onion, spinach), soy, tea (black and green) and red wine (Manach et al., 2004).

There is a bidirectional relation between polyphenols and gut microbiota. The polyphenols’ bioavailability is increased by the microbiota, while unabsorbed polyphenols are involved in maintaining the equilibrium of microbiota in the gut. Suggested mechanisms imply protection against gastro-intestinal disorders and pathogens, strengthening intestinal epithelial tight cell junctions, increasing mucus secretion, stimulating cytokines and modulating the immune response (Ozdal et al., 2016).

From polyphenols class, quercetin is one of the most studied compounds in relationship with gut microbiota. It is a polyphenol derived from plants and belongs to flavonols, a subclass of flavonoid compounds. It can be found in apples, berries, grapes, onions, tea, tomatoes, seed and nuts, but also in medicinal botanicals like *Hypericum perforatum, Ginkgo biloba* and *Sambucus Canadensis* (Li et al., 2016).

It was demonstrated that quercetin has a modulatory role of gut microbiota when the overweight animals were fed with high-fat sucrose diet. Furthermore, quercetin ameliorates the *Firmicutes/Bacteroidetes* ratio in high-fat sucrose diet-fed rats, by increasing the titer of *Bacteroides vulgatus* and *Akkermansia muciniphila*, which have been inversely correlated to obesity. Moreover, it can decrease the titer of *Eubacterium cylindroides* and *Bilophila wadsworthia*, bacteria associated to diet-induced obesity (Etxeberria et al., 2015).

Also, tea, one of the most commonly consumed beverages, is manufactured from the young leaves of the *Camellia sinensis* plant. In today’s society, green or black tea is usually consumed (Cabrera, Giménez & López, 2003). These contain high concentrations of flavanols (epicatechin and catechin) and their esters. High levels of unabsorbed compounds of tea remain in the gut and play an important role in the intestine’s health. Caffeic acid, for example, inhibits the growth of many intestinal pathogenic bacteria, such as *E. coli, Salmonella, Pseudomonas, Clostridium, Bacteroides* (Lee et al., 2006). The main active compounds in green tea are polyphenols. They have important antioxidant and anti-inflammatory effects and influence the activity of NF-kB (nuclear factor kappa B), COX-2 (cyclooxygenase-2) and level of IL-2 (interleukin-2) (Oz, Chen & Villiers, 2013). On the other hand, black tea has several metabolites such as benzoic, phenylacetic and phenylpropionic acids with antimicrobial properties (Duynhoven et al., 2013).

*Lactobacillus plantarum* and *Bacillus subtilis* have a role in the metabolization of theaflavin in active compounds such as gallic acid, pyrogallol, mono gallate and gallate (Chen et al., 2012). There are studies which demonstrate that phenols and their derivates from tea inhibited the growth of pathogenic bacteria (*Clostridium perfringens, Clostridium difficile or Bacteroides spp*) while commensal bacteria (*Lactobacillus, Bifidobacterium*) were less affected. Therefore, phenolic compounds from tea modulate the gut bacteria and can act as a probiotic (Lee et al., 2006).

Resveratrol is a dietary polyphenol and as food supplement is mostly used by patients who suffer from cardiovascular diseases (Bonnefont-Rousselot, 2016; Imamura et al., 2017; Wahab et al., 2017). In an experimental study, it was demonstrated that resveratrol modulates the gut microbiota dysbiosis induced by high-fat diet. Thus, it stimulates the growing of *Lactobacillus* and *Bifidobacterium*, increases the *Bacteroidetes/Firmicutes* ratio and inhibits the growing of *Enterococcus faecalis* (Larrosa et al., 2009; Qiao et al., 2014).

Oregano vulgare is an aromatic plant with multiple phyto-therapeutic uses. Carvacrol and thymol are two main phenols from oregano and they have antimicrobial activity (e.g., on *E. coli*) (Fournomiti et al., 2015; Lopez-Romero et al., 2015). In an experimental study on pigs, it was demonstrated that oregano oil has protective effects against villous atrophy and epithelium cell necrosis and in the same time it decreases the seric endotoxin levels (Zou et al., 2016).

**L-Glutamine (GLN)** is a well-known amino acid which plays an important role in the gut and has an important contribution to generating energy. It is degraded by enterocytes and intestinal luminal bacteria and oxidized by the Krebs cycle, forming ATP (Wang et al., 2014).

In a double-blind study by De Souza et al. (2015) volunteers with body mass index (BMI) over 25 kg/m^2^ were enrolled and divided in two branches, one received GLN and the second one l-alanine (ALA). At the end of the study, there were 33 subjects, 21 in the branch treated with GLN and 12 in that with ALA. The gut microbiota was analyzed before and after 14- day treatment. The results showed that GLN determines changes in the composition of the gut microbiota and the ratio *Firmicutes/Bacteroides* decreased after GLN supplementation, mainly because *Firmicutes* were significantly reduced after GLN treatment. This ratio represents a good biomarker for obesity (De Souza et al., 2015).

In another study, Ren et al. (2014) used 100 mice, divided in two groups, in order to study the effects of dietary supplementation with 1 % L-glutamine for 14 days on the abundance of intestinal bacteria and the activation of intestinal innate immunity in mice. The first group was fed with a normal diet and the second one with normal diet supplemented with 1% GLN for 2 weeks. After this period, the animals were killed and the luminal contents in the jejunum and ileum were collected for analysis. Results showed that the effects of GLN on the abundance of intestinal bacteria were different among different parts of the intestine. Thus, *Firmicutes* were lower in GLN group than in control group in jejunum and ileum, but *Bacteroidetes* were comparable in both groups for these parts of the intestine. On the other hand, *Streptococcus* and *Bifidobacterium* had a high abundance in jejunum, while *Lactobacillus* was not affected. *Streptococcus* and *Lactobacillus* had comparable abundance in ileum in both investigated groups (GLN and control). The authors concluded that introduction of GLN in the mice’s diet changes the intestinal bacteria community and increases the intestinal immunity by affecting the NF-kB (nuclear factor kappa-light-chain-enhancer of activated B cells), MAPK (mitogen-activated protein kinase) and PI3K-Akt (phosphatidylinositol-3-kinases-Protein kinase B) signaling pathways (Ren et al., 2014).

**Omega -3 and -6 fatty acids** are the most used supplements in dyslipidemia, especially in hypertriglyceridemia.

Lui and coworkers studied the effect of omega fatty acids on changes induced in gut microbiota composition. For this purpose, 47 male mice were divided into three groups. Every group was fed with different types of diets rich in saturated fatty acids (SFAs), omega-3 polyunsaturated fatty acids (n −3 PUFAs) and omega-6 polyunsaturated fatty acids (n −6 PUFAs), respectively. The duration of the experiment was 14 weeks and the feces were collected at the beginning and at the end of the experiment. The observed effect on microbiota’s composition was represented by a significant decrease of *Bacteroidetes/Firmicutes* ratio in SFA-rich diet compared with the PUFA-rich diet. Thus, the mice fed with SFA-rich diet acquired a microbial profile similar to those of obese animals (Liu et al., 2012).

A study by Pusceddu and coworkers evaluated the role of n-3 PUFAs on the regulation of gut microbiota in early-life stress (maternal separation). Both non-separable (NS) and maternally separated (MS) groups were divided into three subgroups and every subgroup received saline, low doses of eicosapentaenoic acid (EPA)/docosahexaenoic acid (DHA) (80% EPA, 20% DHA) (0.4g/Kg/day) and high doses of EPA/DHA (1g/Kg/day) respectively. The analysis of microbiota showed microbial dysbiosis in MS saline group, while a high dose of EPA/DHA was associated with a high level of *Butyrivibrio* (butyrate producing bacteria). Moreover, EPA/DHA restored the composition of the gut microbiota in MS rats regardless of the dose (Pusceddu et al., 2015).

Fish oil is one of the most used dietary supplements with a high content of n-3 PUFAs. Yu et al. evaluated in one of their studies whether this nutraceutical product (fish oil) has any effect on gut microbiota. The study used small animals (mice). These were divided into three groups: first received saline, the second received 5 mg/Kg fish oil and the third group 10 mg/Kg fish oil. The results demonstrated that fish oil inhibits the growth of *Helicobacter, Clostridiales, Sphingomonadales, Firmicutes, Pseudomonas sp*. and several uncultured bacteria. It is known that *Helicobacter pylori* plays an important role in the pathogenesis of ulcer, while *Firmicutes* are involved in obesity (Yu et al., 2014). Therefore, the importance of these results resides in the possibility of this supplement to become an important agent in the prophylaxis and treatment of ulcer disease and obesity.

**Berberine**, an alkaloid with quaternary ammonium structure, isolated from *Rhizoma coptidis* or *Berberis sp*., modulates the gut microbiota and has antimicrobial activity on *Firmicutes* and *Bacteroidetes*. Therefore, berberine may contribute to the increase of intestinal gene expression of Fiaf (fasting-induced adipose factor) in mice, which acts as a lipoprotein lipase inhibitor (Zhang et al., 2015; Xu et al., 2017).

For berberine, several important activities have been reported so far, e.g., cytostatic, antiproliferative and antioxidant properties. The alkaloid is considered an “antibiotic with broad spectrum” which may increase *Bacteroides* and decrease *Ruminococcus* in the terminal ileum and colon (Guo et al., 2016).

**Table 2:** The influence of nutraceuticals on microbiota.

Nutraceuticals		Changes in microbiota’s diversity	Reference
Prebiotics	Soy beans and lactose	↑*Bifidobacteria*, *Lactobacillus*	Sangwan et al. (2011)
	Inulin and FOS	↑*Bifidobacteria*, *Lactobacillus*	Velasco et al. (2012)
	Garlic	↓*Bifidobacterium longum , Clostridium nexile*, *E. coli*, *Salmonella typhimuriu, Neisseria gonorrhoeae*, *Staphylococcus aureus*, *Enterococcus faecalis*	Filocamo et al. (2012), Santhosha, Jamuna & Prabhavathi (2013)
	Aloe vera	↓*C. jejuni, C. perfringens, S.aureus, Salmonella, E. coli*	Kim et al. (2014), Cuvas-Limón et al. (2016) and Chiodelli et al. (2017)
Phytoestrogens	Flavonoids	↑*Akkermansia*	Cassidy & Minihane (2017)
	Anthocyanins	↑*Bacteroidetes*, *Actinobacteria*	Overall et al. (2017)
Polyphenols	Quercetin	↑*Bacteroides vulgatus, Akkermansia muciniphila*↓*Eubacterium cylindroides, Bilophila wadsworthia*	Etxeberria et al. (2015)
	Tea from *Camellia sinensis* leaves	↓*E. coli, Salmonella, Pseudomonas, Clostridium, Bacteroides, Clostridium perfringens, Clostridium difficile*	Lee et al. (2006)
	Resveratrol	↑*Lactobacillus,Bifidobacterium*↑*Bacteroidetes/Firmicutes* ratio ↓*Enterococcus faecalis*	Larrosa et al. (2009), Qiao et al. (2014)
Amino acid	L-Glutamine	↓*Firmicutes*↓*Firmicutes/Bacteroides* ratio	De Souza et al. (2015)
Fatty acids	Omega -3 and -6 fatty acids	↓*Bacteroidetes/Firmicutes* ratio ↓*Helicobacter, Clostridiales, Sphingomonadales, Firmicutes, Pseudomonas sp*.	Liu et al. (2012), Yu et al. (2014)
Alkaloids	Berberine	↓*Firmicutes*, *Bacteroidetes, Ruminococcus*	Zhang et al. (2015), Guo et al. (2016)
Algae	Spirulina	↓*Staphulococcus aureus, Bacillus subtilis, E. coli, Pseudomonas aeruginosa*	Beheshtipour et al. (2013), Finamore et al. (2017)
Curcuminoids	Curcumin	↑*Lactobacillus*↓*Coriobacteriales*	Ramalingam et al. (2016).

**Spirulina** (*Arthrospira platensis*), a cyanobacteria (blue–green algae), mostly used as a food supplement, has the capacity to inhibit the growth of Gram positive and Gram negative bacteria (*Staphulococcus aureus, Bacillus subtilis, E. coli, Pseudomonas aeruginosa* etc.). This antibacterial effect is due to an extracellular metabolite produced by spirulina. In addition, the microbiota’s modulatory effect of spirulina was attributed to the active compounds found in this plant (glutamate, aspartate, carbohydrates or phenolic compounds). These substances have a well-known antimicrobialand bacteriostatic effect and the capacity to stimulate the growth of probiotics (Beheshtipour et al., 2013; Finamore et al., 2017). Due to this effect, the spirulina biomass can become a natural product which could be added to fermented milk to increase the production of *Lactobacillus* and also the number of viable cells which reach the intestine (Bhowmik, Dubey & Mehra, 2009).

**Curcumin** is a bioactive component extracted from the *Curcuma longa* plant. In an experimental model of inflammatory bowel disease on small animals (mice) the supplementation of the diet with curcumin determined a significant change in gut microbiota: increasing the growth of *Lactobacillus* and decreasing the *Coriobacteriales* (Ramalingam et al., 2016).

## Conclusions

This review attempted to underline the close link between microbiota and the most used nutraceuticals (Table 2). Thus, there are compounds, such as those mentioned above, which are nutraceuticals with real observed benefits on human health. In the past years, more and more patients have been using nutraceuticals without a medical recommendation, many of these being OTC (Over The Counter). Patients are not aware neither of the benefits nor of the risks of nutraceuticals, therefore the population could really benefit from a thorough education on this subject. Imbalance of microbiota is involved in many diseases, it can increase their risk of appearance, therefore it is critical to have a good knowledge of the main nutraceuticals in use and establish in what way they affect the composition of microbiota.

The changes in the gut microbiota are being accepted as important elements in the development of many diseases. Many nutraceuticals (prebiotics, probiotics, omega 3 acids, aloe vera etc.) may restore microbial homeostasis, reduce the negative effects of pathogenic agents, influence the inflammation pathway or increase the effect of standard allopathic therapy. Based on data published, it can be concluded that nutraceuticals mentioned above have promising clinical results and are likely to have a beneficial effect.

However, further studies are needed to complete the assessment of nutraceuticals in health’s benefit as a consequence of microbiota’s changing.

## Additional Information and Declarations

### Data Availability

The following information was supplied regarding data availability: The research in this article did not generate any data or code (it is a literature review).

## References

[ref-1] Alex S, Lange K, Amolo T, Grinstead JS, Haakonsson AK, Szalowska E, Koppen A, Mudde K, Haenen D, Al-Lahham S, Roelofsen H, Houtman R, Van der Burg B, Mandrup S, Bonvin AMJJ, Kalkhoven E, Muller M, Hooiveld GJ, Kersten S (2013). Short-chain fatty acids stimulate angiopoietin-like 4 synthesis in human colon adenocarcinoma cells by activating peroxisome proliferator-activated receptor. Molecular and Cellular Biology.

[ref-2] Alisi A, Bedogni G, Baviera G, Giorgio V, Porro E, Paris C, Giammaria P, Reali L, Anania F, Nobili V (2014). Randomised clinical trial: the beneficial effects of VSL#3 in obese children with non-alcoholic steatohepatitis. Alimentary Pharmacology and Therapeutics.

[ref-3] Alvarez-Curto E, Milligan G (2016). Metabolism meets immunity: the role of free fatty acid receptors in the immune system. Biochemical Pharmacology.

[ref-4] Beheshtipour H, Mortazavian AM, Mohammadi R, Sohrabvandi S, Khosravi-Darani K (2013). Supplementation of spirulina platensis and chlorella vulgaris algae into probiotic fermented milks. Comprehensive Reviews in Food Science and Food Safety.

[ref-5] Bhowmik D, Dubey J, Mehra S (2009). Probiotic efficiency of spirulina platensis -stimulating growth of lactic acid bacteria. World Journal of Dairy & Food Sciences.

[ref-6] Bilal I, Chowdhury A, Davidson J, Whitehead S (2014). Phytoestrogens and prevention of breast cancer: the contentious debate. World Journal of Clinical Oncology.

[ref-7] Bindels LB, Neyrinck AM, Salazar N, Taminiau B, Druart C, Muccioli GG, François E, Blecker C, Richel A, Daube G, Mahillon J, De Los Reyes-Gavilán CG, Cani PD, Delzenne NM (2015). Non digestible oligosaccharides modulate the gut microbiota to control the development of leukemia and associated cachexia in mice. PLOS ONE.

[ref-8] Blanck HM, Bowman BA, Cooper GR, Myers L, Miller DT (2003). Biomarkers of nutritional exposure and nutritional status laboratory issues: use of nutritional biomarkers 1. Environmental Health.

[ref-9] Bonnefont-Rousselot D (2016). Resveratrol and cardiovascular diseases. Nutrients.

[ref-10] Bron PA, Kleerebezem M, Brummer R-J, Cani PD, Mercenier A, Macdonald TT, Garcia-Ródenas CL, Wells JM (2017). Can probiotics modulate human disease by impacting intestinal barrier function?. The British Journal of Nutrition.

[ref-11] Buford TW (2017). (Dis)Trust your gut: the gut microbiome in age-related inflammation, health, and disease. Microbiome.

[ref-12] Cabrera C, Giménez R, López MC (2003). Determination of tea components with antioxidant activity. Journal of Agricultural and Food Chemistry.

[ref-13] Carding S, Verbeke K, Vipond DT, Corfe BM, Owen LJ (2015). Dysbiosis of the gut microbiota in disease. Microbial Ecology in Health & Disease.

[ref-14] Cassidy A, Minihane AM (2017). The role of metabolism (and the microbiome) in defining the clinical efficacy of dietary flavonoids. American Journal of Clinical Nutrition.

[ref-15] Chaiyasut C, Pengkumsri N, Sirilun S, Peerajan S, Khongtan S, Sivamaruthi BS (2017). Assessment of changes in the content of anthocyanins, phenolic acids, and antioxidant property of Saccharomyces cerevisiae mediated fermented black rice bran. AMB Express.

[ref-16] Chen H, Hayek S, Rivera Guzman J, Gillitt ND, Ibrahim SA, Jobin C, Sang S (2012). The microbiota is essential for the generation of black tea theaflavins-derived metabolites. PLOS ONE.

[ref-17] Chevalier C, Stojanović O, Colin DJ, Suarez-Zamorano N, Tarallo V, Veyrat-Durebex C, Rigo D, Fabbiano S, Stevanović A, Hagemann S, Montet X, Seimbille Y, Zamboni N, Hapfelmeier S, Trajkovski M (2015). Gut microbiota orchestrates energy homeostasis during cold. Cell.

[ref-18] Chiodelli G, Pellizzoni M, Ruzickova G, Lucini L (2017). Effect of different aloe fractions on the growth of lactic acid bacteria. Journal of Food Science.

[ref-19] Compare D, Coccoli P, Rocco A, Nardone OM, De Maria S, Carten M, Nardone G (2012). Gut-liver axis: the impact of gut microbiota on non alcoholic fatty liver disease. Nutrition, Metabolism and Cardiovascular Diseases.

[ref-20] Cuvas-Limón R, Julio M, Carlos C, Mario C, Mussatto S, Ruth B-C (2016). Aloe vera and probiotics: a new alternative to symbiotic functional foods. Annual Research & Review in Biology.

[ref-21] Dahiya DK, Puniya M, Shandilya UK, Dhewa T (2017). Gut microbiota modulation and its relationship with obesity using prebiotic fibers and probiotics: a review. Frontiers in Microbiology.

[ref-22] Das L, Bhaumik E, Raychaudhuri U, Chakraborty R (2012). Role of nutraceuticals in human health. Journal of Food Science and Technology.

[ref-23] De Angelis M, Piccolo M, Vannini L, Siragusa S, De Giacomo A, Serrazzanetti DI, Cristofori F, Guerzoni ME, Gobbetti M, Francavilla R (2013). Fecal microbiota and metabolome of children with autism and pervasive developmental disorder not otherwise specified. PLOS ONE.

[ref-24] De Goffau MC, Fuentes S, Van Den Bogert B, Honkanen H, De Vos WM, Welling GW, Hyöty H, Harmsen HJM (2014). Aberrant gut microbiota composition at the onset of type 1 diabetes in young children. Diabetologia.

[ref-25] Den Besten G, Van Eunen K, Groen AK, Venema K, Reijngoud D-J, Bakker BM (2013). The role of short-chain fatty acids in the interplay between diet, gut microbiota, and host energy metabolism. The Journal of Lipid Research.

[ref-26] Derrien M, Vaughan EE, Plugge CM, De Vos WM (2004). *Akkermansia muciniphila* gen. nov., sp. nov., a human intestinal mucin-degrading bacterium. International Journal of Systematic and Evolutionary Microbiology.

[ref-27] De Souza AZZ, Zambom AZ, Abboud KY, Reis SK, Tannihão F, Guadagnini D, Saad MJA, Prada PO (2015). Oral supplementation with l-glutamine alters gut microbiota of obese and overweight adults: a pilot study. Nutrition.

[ref-28] Dupont HL (2014). Review article: evidence for the role of gut microbiota in irritable bowel syndrome and its potential influence on therapeutic targets. Alimentary Pharmacology and Therapeutics.

[ref-29] Duynhoven J Van, Vaughan EE, Dorsten F Van, Gomez-roldan V, Vos R De, Vervoort J, Hooft JJJ Van Der, Roger L, Draijer R, Jacobs DM (2013). Interactions of black tea polyphenols with human gut microbiota: implications for gut and cardiovascular health.

[ref-30] Elderman M, Sovran B, Hugenholtz F, Graversen K, Huijskes M, Houtsma E, Belzer C, Boekschoten M, Vos P De, Dekker J, Wells J, Faas M (2017). The effect of age on the intestinal mucus thickness, microbiota composition and immunity in relation to sex in mice. PLOS ONE.

[ref-31] Espín JC, González-Sarrías A, Tomás-Barberán FA (2017). The gut microbiota: a key factor in the therapeutic effects of (poly)phenols. Biochemical Pharmacology.

[ref-32] Etxeberria U, Arias N, Boqué N, Macarulla MT, Portillo MP, Martínez JA, Milagro FI (2015). Reshaping faecal gut microbiota composition by the intake of trans-resveratrol and quercetin in high-fat sucrose diet-fed rats. Journal of Nutritional Biochemistry.

[ref-33] Festi D, Schiumerini R, Eusebi LH, Marasco G, Taddia M, Colecchia A (2014). Gut microbiota and metabolic syndrome. World Journal of Gastroenterology.

[ref-34] Filocamo A, Nueno-Palop C, Bisignano C, Mandalari G, Narbad A (2012). Effect of garlic powder on the growth of commensal bacteria from the gastrointestinal tract. Phytomedicine.

[ref-35] Finamore A, Palmery M, Bensehaila S, Peluso I (2017). Antioxidant, immunomodulating, and microbial-modulating activities of the sustainable and ecofriendly spirulina. Oxidative Medicine and Cellular Longevity.

[ref-36] Fournomiti M, Kimbaris A, Mantzourani I, Plessas S, Theodoridou I, Papaemmanouil V, Kapsiotis I, Panopoulou M, Stavropoulou E, Bezirtzoglou EE, Alexopoulos A (2015). Antimicrobial activity of essential oils of cultivated oregano (Origanum vulgare), sage (Salvia officinalis), and thyme (Thymus vulgaris) against clinical isolates of *Escherichia coli*, Klebsiella oxytoca, and Klebsiella pneumoniae. Microbial Ecology in Health and Disease.

[ref-37] Franke AA, Lai JF, Halm BM, States U (2014). Absorption, distribution, metabolism, and excretion of isoflavonoids after soy intake. Archives of Biochemistry and Biophysics.

[ref-38] Gaya P, Medina M, Sánchez-Jiménez A, Landete J (2016). Phytoestrogen metabolism by adult human gut microbiota. Molecules.

[ref-39] Guadamuro L, Dohrmann AB, Tebbe CC, Mayo B, Delgado S (2017). Bacterial communities and metabolic activity of faecal cultures from equol producer and non-producer menopausal women under treatment with soy isoflavones. BMC Microbiology.

[ref-40] Guo Y, Zhang Y, Huang W, Selwyn FP, Klaassen CD (2016). Dose–response effect of berberine on bile acid profile and gut microbiota in mice. BMC Complementary and Alternative Medicine.

[ref-41] Gupta S, Chauhan D, Mehla K, Sood P, Nair A (2010). An overview of nutraceuticals: current scenario. Journal of Basic and Clinical Pharmacy.

[ref-42] Hara T, Hirasawa A, Ichimura A, Kimura I, Tsujimoto G (2011). Free fatty acid receptors FFAR1 and GPR120 as novel therapeutic targets for metabolic disorders. Journal of Pharmaceutical Sciences.

[ref-43] Hua X, Goedert JJ, Pu A, Yu G, Shi J (2016). Allergy associations with the adult fecal microbiota: analysis of the American Gut Project. EBioMedicine.

[ref-44] Huazano-Garcia A, Hakdong S, Lopez G (2017). Modulation of gut microbiota of overweight mice by agavins and their association with body weight. Nutrients.

[ref-45] Imamura H, Yamaguchi T, Nagayama D, Saiki A, Shirai K, Tatsuno I (2017). Resveratrol Ameliorates arterial stiffness assessed by cardio-ankle vascular index in patients with type 2 diabetes mellitus. International Heart Journal.

[ref-46] Iranshahi M, Rezaee R, Parhiz H, Roohbakhsh A, Soltani F (2015). Protective effects of flavonoids against microbes and toxins: the cases of hesperidin and hesperetin. Life Sciences.

[ref-47] Jandhyala SM, Talukdar R, Subramanyam C, Vuyyuru H, Sasikala M, Reddy DN (2015). Role of the normal gut microbiota. World Journal of Gastroenterology.

[ref-48] Jarošová B, Javůrek J, Adamovský O, Hilscherová K (2015). Phytoestrogens and mycoestrogens in surface waters—their sources, occurrence, and potential contribution to estrogenic activity. Environment International.

[ref-49] Johnston BC, Goldenberg JZ, Vandvik PO, Sun X, Guyatt GH (2011). Probiotics for the prevention of pediatric antibiotic-associated diarrhea. Cochrane Database of Systematic Reviews.

[ref-50] Joseph J, Depp C, Shih PAB, Cadenhead KS, Schmid-Schönbein G (2017). Modified mediterranean diet for enrichment of short chain fatty acids: potential adjunctive therapeutic to target immune and metabolic dysfunction in schizophrenia?. Frontiers in Neuroscience.

[ref-51] Kim CH, Park J, Kim M (2014). Gut microbiota-derived short-chain fatty acids, T cells, and inflammation. Immune Network.

[ref-52] Kim YW, Jeong YJ, Kim AY, Son HH, Lee JA, Jung CH, Kim CH, Kim J (2014). Lactobacillus brevis strains from fermented Aloe vera survive gastroduodenal environment and suppress common food borne enteropathogens. PLOS ONE.

[ref-53] Kirjavainen PV, Arvola T, Salminen SJ, Isolauri E (2002). Aberrant composition of gut microbiota of allergic infants: a target of bifidobacterial therapy at weaning?. Gut.

[ref-54] Kolida S, Tuohy K, Gibson GR (2002). Prebiotic effects of inulin and oligofructose. British Journal of Nutrition.

[ref-55] Kumar M, Nagpal R, Kumar R, Hemalatha R, Verma V, Kumar A, Chakraborty C, Singh B, Marotta F, Jain S, Yadav H (2012). Cholesterol-lowering probiotics as potential biotherapeutics for metabolic diseases. Experimental Diabetes Research.

[ref-56] Laparra JM, Sanz Y (2010). Interactions of gut microbiota with functional food components and nutraceuticals. Pharmacological Research.

[ref-57] Larrosa M, Yañéz Gascón MJ, Selma MV, González-Sarrías A, Toti S, Cerón JJ, Tomás-Barberán F, Dolara P, Espín JC (2009). Effect of a low dose of dietary resveratrol on colon microbiota, inflammation and tissue damage in a DSS-induced colitis rat model. Journal of Agricultural and Food Chemistry.

[ref-58] Lee HC, Jenner AM, Low CS, Lee YK (2006). Effect of tea phenolics and their aromatic fecal bacterial metabolites on intestinal microbiota. Research in Microbiology.

[ref-59] Levy M, Blacher E, Elinav E (2017). Microbiome, metabolites and host immunity. Current Opinion in Microbiology.

[ref-60] Li Y, Yao J, Han C, Yang J, Chaudhry MT, Wang S, Liu H, Yin Y (2016). Quercetin, inflammation and immunity. Nutrients.

[ref-61] Li G, Young KD (2013). Indole production by the tryptophanase TnaA in *escherichia coli* is determined by the amount of exogenous tryptophan. Microbiology.

[ref-62] Liu T, Hougen H, Vollmer AC, Hiebert SM (2012). Gut bacteria profiles of Mus musculus at the phylum and family levels are influenced by saturation of dietary fatty acids. Anaerobe.

[ref-63] Lopez-Romero JC, González-Ríos H, Borges A, Simões M (2015). Antibacterial effects and mode of action of selected essential oils components against *escherichia coli* and staphylococcus aureus. Evidence-Based Complementary and Alternative Medicine.

[ref-64] Louis P, Flint HJ (2017). Formation of propionate and butyrate by the human colonic microbiota. Environmental Microbiology.

[ref-65] Lucas López R, Grande Burgos MJ, Gálvez A, Pérez Pulido R (2017). The human gastrointestinal tract and oral microbiota in inflammatory bowel disease: a state of the science review. Apmis.

[ref-66] Machado MV, Cortez-Pinto H (2012). Gut microbiota and nonalcoholic fatty liver disease. Annals of Hepatology.

[ref-67] Manach C, Scalbert A, Morand C, Rémésy C, Jiménez L (2004). Polyphenols: food sources and bioavailability. The American Journal of Clinical Nutrition.

[ref-68] Marlene R (2013). Abundance and diversity of microbiota in type 2 diabetes and obesity. Journal of Diabetes & Metabolism.

[ref-69] McFarlin BK, Henning AL, Bowman EM, Gary MA, Carbajal KM (2017). Oral spore-based probiotic supplementation was associated with reduced incidence of post-prandial dietary endotoxin, triglycerides, and disease risk biomarkers. World Journal of Gastrointestinal Pathophysiology.

[ref-70] Montalto M, D’Onofrio F, Gallo A, Cazzato A, Gasbarrini G (2009). Intestinal microbiota and its functions. Digestive and Liver Disease Supplements.

[ref-71] Morrison DJ, Preston T (2016). Formation of short chain fatty acids by the gut microbiota and their impact on human metabolism. Gut Microbes.

[ref-72] Murri M, Leiva I, Gomez-Zumaquero JM, Tinahones FJ, Cardona F, Soriguer F, Queipo-Ortuño MI (2013). Gut microbiota in children with type 1 diabetes differs from that in healthy children: a case-control study. BMC Medicine.

[ref-73] Ottman N, Reunanen J, Meijerink M, Pietila TE, Kainulainen V, Klievink J, Huuskonen L, Aalvink S, Skurnik M, Boeren S, Satokari R, Mercenier A, Palva A (2017). Pili-like proteins of *Akkermansia muciniphila* modulate host immune responses and gut barrier function. PLOS ONE.

[ref-74] Overall J, Bonney SA, Wilson M, Beermann A, Grace MH, Esposito D, Lila MA, Komarnytsky S (2017). Metabolic effects of berries with structurally diverse anthocyanins. International Journal of Molecular Sciences.

[ref-75] Oz HS, Chen T, De Villiers WJS (2013). Green tea polyphenols and sulfasalazine have parallel anti-inflammatory properties in colitis models. Frontiers in Immunology.

[ref-76] Ozdal T, Sela DA, Xiao J, Boyacioglu D, Chen F, Capanoglu E (2016). The reciprocal interactions between polyphenols and gut microbiota and effects on bioaccessibility. Nutrients.

[ref-77] Pandey M, Verma RK, Saraf SA (2010). Nutraceuticals: new era of medicine and health. Asian Journal of Pharmaceutical and Clinical Research.

[ref-78] Park M, Kwon B, Ku S, Ji G (2017). The efficacy of bifidobacterium longum BORI and lactobacillus acidophilus AD031 probiotic treatment in infants with rotavirus infection. Nutrients.

[ref-79] Parracho HMRT, Bingham MO, Gibson GR, McCartney AL (2005). Differences between the gut microflora of children with autistic spectrum disorders and that of healthy children. Journal of Medical Microbiology.

[ref-80] Peterson J, Dwyer J, Adlercreutz H, Scalbert A, Mccullough ML (2011). Dietary lignans: physiology and potential for cardiovascular disease risk reduction. Nutrition Reviews.

[ref-81] Pluznick J (2014). A novel SCFA receptor, the microbiota, and blood pressure regulation. Gut Microbes.

[ref-82] Pusceddu MM, El Aidy S, Crispie F, O’Sullivan O, Cotter P, Stanton C, Kelly P, Cryan JF, Dinan TG (2015). N-3 polyunsaturated fatty acids (PUFAs) reverse the impact of early-life stress on the gut microbiota. PLOS ONE.

[ref-83] Qiao Y, Sun J, Xia S, Tang X, Shi Y, Le G (2014). Effects of resveratrol on gut microbiota and fat storage in a mouse model with high-fat-induced obesity. Food & Function.

[ref-84] Ramakrishna BS (2013). Role of the gut microbiota in human nutrition and metabolism. Journal of Gastroenterology and Hepatology.

[ref-85] Ramalingam R, Harrison CA, Besselsen DG, John H (2016). The role of curcumin in modulating colonic microbiota during colitis and colon cancer prevention. Inflammatory Bowel Diseases.

[ref-86] Ren W, Duan J, Yin J, Liu G, Cao Z, Xiong X, Chen S, Li T, Yin Y, Hou Y, Wu G (2014). Dietary l-glutamine supplementation modulates microbial community and activates innate immunity in the mouse intestine. Amino Acids.

[ref-87] Riva A, Togni S, Giacomelli L, Franceschi F, Eggenhoffner R, Feragalli B, Belcaro G, Cacchio M, Shu H, Dugall M (2017). Effects of a curcumin-based supplementation in asymptomatic subjects with low bone density: a preliminary 24-week supplement study. European Review for Medical and Pharmacological Sciences.

[ref-88] Rodríguez Galdón B, Rodríguez Rodríguez EM, Díaz Romero C (2008). Flavonoids in onion cultivars (Allium cepa L.). Journal of Food Science.

[ref-89] Romano M, Vitaglione P, Sellitto S, D’Argenio G (2012). Nutraceuticals for protection and healing of gastrointestinal mucosa. Current Medicinal Chemistry.

[ref-90] Sangwan V, Tomar SK, Singh RRB, Singh AK, Ali B (2011). Galactooligosaccharides: novel components of designer foods. Journal of Food Science.

[ref-91] Santhosha SG, Jamuna P, Prabhavathi SN (2013). Bioactive components of garlic and their physiological role in health maintenance: a review. Food Bioscience.

[ref-92] Schneeberger M, Everard A, Gómez-Valadés AG, Matamoros S, Ramírez S, Delzenne NM, Gomis R, Claret M, Cani PD (2015). *Akkermansia muciniphila* inversely correlates with the onset of inflammation, altered adipose tissue metabolism and metabolic disorders during obesity in mice. Scientific Reports.

[ref-93] Schwiertz A, Taras D, Schäfer K, Beijer S, Bos NA, Donus C, Hardt PD (2010). Microbiota and SCFA in lean and overweight healthy subjects. Obesity.

[ref-94] Shashkova T, Popenko A, Tyakht A, Peskov K, Kosinsky Y (2016). Agent based modeling of human gut microbiome interactions and perturbations. PLOS ONE.

[ref-95] Sirotkin AV, Harrath AH (2014). Phytoestrogens and their effects. European Journal of Pharmacology.

[ref-96] Steinmeyer S, Lee K, Jayaraman A, Alaniz RC (2015). Microbiota metabolite regulation of host immune homeostasis: a mechanistic missing link. Current Allergy and Asthma Reports.

[ref-97] Tang WHW, Hazen SL (2014). The contributory role of gut microbiota in cardiovascular disease. Journal of Clinical Investigation.

[ref-98] Thursby E, Juge N (2017). Introduction to the human gut microbiota. Biochemical Journal.

[ref-99] Tremaroli V, Bäckhed F (2012). Functional interactions between the gut microbiota and host metabolism. Nature.

[ref-100] Trøseid M (2017). Gut microbiota and acute coronary syndromes: ready for use in the emergency room?. European Heart Journal.

[ref-101] Vandenplas Y, Zakharova I, Dmitrieva Y (2017). Oligosaccharides in infant formula: more evidence to validate the role of prebiotics. British Journal of Nutrition.

[ref-102] Velasco C, Lozano MA, Moreno Y, Paron L, Cuerda C De, Bretón I (2012). Effect of a mixture of inulin and fructo-oligosaccharide on lactobacillus and bifidobacterium intestinal microbiota of patients receiving radiotherapy; a randomised, double-blind, placebo-controlled trial. Nutrición Hospitalaria.

[ref-103] Vitale DC, Piazza C, Melilli B, Drago F, Salomone S (2013). Isoflavones: estrogenic activity, biological effect and bioavailability. European Journal of Drug Metabolism and Pharmacokinetics.

[ref-104] Wahab A, Gao K, Jia C, Zhang F, Tian G, Murtaza G, Chen J (2017). Significance of resveratrol in clinical management of chronic diseases. Molecules.

[ref-105] Walker AW, Lawley TD (2013). Therapeutic modulation of intestinal dysbiosis. Pharmacological Research.

[ref-106] Wang B, Wu G, Zhou Z, Dai Z, Sun Y, Ji Y, Li W, Wang W, Liu C, Han F, Wu Z (2014). Glutamine and intestinal barrier function. Amino Acids.

[ref-107] Weitkunat K, Stuhlmann C, Postel A, Rumberger S, Fankhänel M, Woting A, Petzke KJ, Gohlke S, Schulz TJ, Blaut M, Klaus S, Schumann S (2017). Short-chain fatty acids and inulin, but not guar gum, prevent diet-induced obesity and insulin resistance through differential mechanisms in mice. Scientific Reports.

[ref-108] Wong VW-S, Tse C-H, Lam TT-Y, Wong GL-H, Chim AM-L, Chu WC-W, Yeung DK-W, Law PT-W, Kwan H-S, Yu J, Sung JJ-Y, Chan HL-Y (2013). Molecular characterization of the fecal microbiota in patients with nonalcoholic steatohepatitis—a longitudinal study. PLOS ONE.

[ref-109] Woting A, Blaut M (2016). The intestinal microbiota in metabolic disease. Nutrients.

[ref-110] Wu X, Ma C, Han L, Nawaz M, Gao F, Zhang X, Yu P, Zhao C, Li L, Zhou A, Wang J, Moore JE, Cherie Millar B, Xu J (2010). Molecular characterisation of the faecal microbiota in patients with type II diabetes. Current Microbiology.

[ref-111] Xu J, Liu X, Pan W, Zou D (2017). Berberine protects against diet-induced obesity through regulating metabolic endotoxemia and gut hormone levels. Molecular Medicine Reports.

[ref-112] Yu H-N, Zhu J, Pan W, Shen S-R, Shan W-G, Das UN (2014). Effects of fish oil with a high content of n-3 polyunsaturated fatty acids on mouse gut microbiota. Archives of Medical Research.

[ref-113] Zeng H, Taussig DP, Cheng WH, Johnson LAK, Hakkak R (2017). Butyrate inhibits cancerous HCT116 colon cell proliferation but to a lesser extent in noncancerous NCM460 colon cells. Nutrients.

[ref-114] Zhang X, Zhao Y, Xu J, Xue Z, Zhang M, Pang X, Zhang X, Zhao L (2015). Modulation of gut microbiota by berberine and metformin during the treatment of high-fat diet-induced obesity in rats. Scientific Reports.

[ref-115] Zhou K (2017). Strategies to promote abundance of *Akkermansia muciniphila*, an emerging probiotics in the gut, evidence from dietary intervention studies. Journal of Functional Foods.

[ref-116] Zhu L, Liu W, Alkhouri R, Baker RD, Bard JE, Quigley EM, Baker SS (2014). Structural changes in the gut microbiome of constipated patients. Physiological Genomics.

[ref-117] Zou Y, Xiang Q, Wang J, Peng J, Wei H (2016). Oregano essential oil improves intestinal morphology and expression of tight junction proteins associated with modulation of selected intestinal bacteria and immune status in a pig model. BioMed Research International.

